# A Machine Learned Classifier That Uses Gene Expression Data to Accurately Predict Estrogen Receptor Status

**DOI:** 10.1371/journal.pone.0082144

**Published:** 2013-12-02

**Authors:** Meysam Bastani, Larissa Vos, Nasimeh Asgarian, Jean Deschenes, Kathryn Graham, John Mackey, Russell Greiner

**Affiliations:** 1 Department of Computing Science, University of Alberta, Edmonton, Alberta, Canada; 2 Department of Oncology, University of Alberta, Edmonton, Alberta, Canada; 3 Alberta Innovates Centre for Machine Learning, Edmonton, Alberta, Canada; 4 Department of Pathology and Laboratory Medicine, University of Alberta, Edmonton, Alberta, Canada; 5 Cross Cancer Institute, Edmonton, Alberta, Canada; University of Glasgow, United Kingdom

## Abstract

**Background:**

Selecting the appropriate treatment for breast cancer requires accurately determining the estrogen receptor (ER) status of the tumor. However, the standard for determining this status, immunohistochemical analysis of formalin-fixed paraffin embedded samples, suffers from numerous technical and reproducibility issues. Assessment of ER-status based on RNA expression can provide more objective, quantitative and reproducible test results.

**Methods:**

To learn a parsimonious RNA-based classifier of hormone receptor status, we applied a machine learning tool to a training dataset of gene expression microarray data obtained from 176 frozen breast tumors, whose ER-status was determined by applying ASCO-CAP guidelines to standardized immunohistochemical testing of formalin fixed tumor.

**Results:**

This produced a three-gene classifier that can predict the ER-status of a novel tumor, with a cross-validation accuracy of 93.17±2.44%. When applied to an independent validation set and to four other public databases, some on different platforms, this classifier obtained over 90% accuracy in each. In addition, we found that this prediction rule separated the patients' recurrence-free survival curves with a hazard ratio lower than the one based on the IHC analysis of ER-status.

**Conclusions:**

Our efficient and parsimonious classifier lends itself to high throughput, highly accurate and low-cost RNA-based assessments of ER-status, suitable for routine high-throughput clinical use. This analytic method provides a proof-of-principle that may be applicable to developing effective RNA-based tests for other biomarkers and conditions.

## Introduction

Invasive breast adenocarcinoma is a common cancer whose clinical management is guided by predictive biomarkers. In particular, clinicians rely on the predictive value of tumor Estrogen Receptor (ER) status to decide whether to apply endocrine therapy.

At present, immunohistochemical (IHC) testing is most frequently used to assign tumor ER-status, where antibodies directed against the ER protein are applied to formalin-fixed, paraffin-embedded tumor samples, and the abundance of ER is determined semi-quantitatively by light microscopy. Those patients with tumors rich in ERs (ER+) are most likely to benefit from endocrine therapy, while those with ER-poor tumors (ER-) typically derive no benefit from endocrine therapy [1]. Consequently, those individuals found to have ER+ disease are offered hormonal therapy, either for prevention of recurrence after definitive surgery, or for tumor suppression in the setting of advanced disease. Those with ER- disease do not receive endocrine therapy, and instead are frequently offered cytotoxic chemotherapy.

The use of IHC for determining ER-status has many limitations, including the lack of a “gold-standard” assay with which to calibrate test results, the difficulties in standardization of several parameters, including pre-analytic variables (warm and cold ischemic times, type of fixative used, duration and quality of tissue fixation), the selection and titration of antibody, antigen retrieval and signal detection methods, the appropriate choice of positive and negative controls, and the standardized interpretation of the results of the IHC assay. Due to these issues, an international expert panel concluded that up to 20% of current IHC determinations of ER-status worldwide may be inaccurate (falsely negative or falsely positive) [2]. The lack of standardization and the complexity of determining IHC ER-status has contributed to widely-reported failures in providing optimal breast cancer care [3]. Consequently, more accurate and less subjective ways to determine tumor ER-status would have clinical value.

Recent advances in bio-profiling technologies have allowed the large scale assessment of multiple biomarkers, including quantitative assessment of RNA with frozen [4] and paraffin-embedded formalin-fixed tissues [5]. To help find a RNA-based test for ER-status, we determined the gene expression levels across the transcriptome in invasive breast tumors from a large cohort of women with known ER-status determined by guideline-standardized IHC, and then applied machine learning technologies to generate a parsimonious effective predictor of ER-status, amenable to high throughput and low cost testing. While our learner had access to the expression levels of all of the genes, it produced a predictor that requires only three gene expression values; this differs from prior classifiers that required determining the expression levels of large numbers of genes [6], [7]. Moreover, we show that our learned predictor works effectively on other datasets, from other labs, some using other platforms.

## Materials and Methods

### Sample Selection

Institutional ethics approval through the Alberta Cancer Research Ethics Committee and patient informed written consent were obtained for collection of surgical specimens, relevant clinical data, and tissue analysis. We used 176 treatment-naive primary breast cancer cases from the Canadian Breast Cancer Foundation Tumor Bank (CBCF TB) as a training set for data analysis, hereafter called the E176 group [8]. A second distinct group of 23 treatment-naive breast tumor samples collected under the same protocol as E176 was obtained from the CBCF TB, referred to as the E23 group, and used as a validation set. All tumor samples were collected at surgery and frozen in liquid nitrogen within 20 min of devitalization. Evaluation of histology slides from tissue adjacent to the frozen samples indicated that at least 70% of the cells present were tumor cells.

The ER-status of each of these primary tumors was determined in a single central laboratory using the clinical standard antibody (Ventana, Tucson, AZ) applied to formalin-fixed paraffin-embedded tissue. We followed the ASCO-CAP guideline [2] methods, considering a tumor as positive whenever at least 1% of the tumor nuclei in the sample were positive, in the presence of expected reactivity of normal epithelial elements and external controls. Samples were scored by a single board certified breast cancer pathologist (JD), blinded to gene expression analysis and clinical outcomes. The results of this analysis were in complete accordance with the ER-status determined by a panel of 7 pathologists during the initial breast cancer diagnosis. We found 63.3% of the E176 group, and 60.9% of the E23 group, were ER+.

### Microarray expression analysis

Total RNA was isolated from the frozen samples using Trizol (Sigma-Aldrich, Oakville, ON, CAN) and purified using Qiagen RNeasy columns (Mississauga, ON, CAN) according to the manufacturer's recommended protocols. The RNA was then quantified using a NanoDrop 1000 Spectrophotometer (NanoDrop Technologies, Wilmington, DE, USA) and its integrity evaluated using a Bioanalyzer 2100 (Agilent Technologies, Santa Clara, CA, USA) according to the manufacturer's protocols. RNA samples with RNA Integrity Numbers (RIN) greater than 7.0 were used in this study.

This RNA was subjected to linear amplification and Cy3 labeling and hybridization to Agilent Whole Human Genome Arrays using Agilent kits (One Color Low RNA Input Linear Amplification Kit Plus, One Color RNA Spike-In Kit and Gene Expression Hybridization Kit) according to the manufacturer's recommended protocols. After the arrays were scanned using an Agilent Scanner, the data was extracted and the quality evaluated using Feature Extraction Software 9.5 (Agilent) [9]. The data was normalized and analyzed using GeneSpring GX 7.3.1 (Agilent).

The data discussed in this publication have been deposited in NCBI's Gene Expression Omnibus [10] and are accessible through GEO Series accession number GSE29210 (http://www.ncbi.nlm.nih.gov/geo/query/acc.cgi?acc=GSE29210). Our studies also used four other GEO datasets: GSE26338 [9], GSE5546 [11], GSE19615 [12] and GSE31448 [13].

### Analytical Tools

We refer to each oligonucleotide in the array as a feature, and note that each gene is represented by one or more features. The value of each feature in each array was [N1] baselined to 0.1 (each value less than 0.1 was replaced with 0.1), [N2] normalized per array (each measurements on each array was divided by the 50^th^ percentile value for that array) and then [N3] normalized per feature (each feature was divided by the median of its measurements in all samples). The N1–N3 normalization steps are performed by the GeneSpring software. We then [N4] transformed the data into z-scores, 
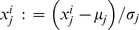
 by subtracting from each feature value 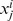 the mean for this j-th feature μ_j_, then dividing by the standard deviation σ_j_ for that feature; hence each transformed feature has zero mean and unit variance over the dataset [14]. Features were then filtered to include only those that were annotated with a GenBank accession number and were present in at least 44 of the E176 training samples; this produced a set of 27,688 features. The resulting dataset 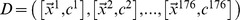 is over the 176 patients and contains 27,688 gene expression values 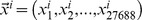 for each patient (i = 1,2,…, 176) as well as each patient's ER-status 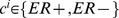, as described above. The E23 validation set was normalized ([N1]–[N4]) independently of the E176 data; that dataset was then filtered to include only the set of 27,688 features used in the E176 analysis.

### Mutual Information (for Biostatistical Analysis)

The standard biostatistics approach to analyzing this microarray gene expression data *D* seeks univariate correlations, with the goal of finding the individual features most relevant to the ER-status outcome. To do this, we estimated the relevance of each feature using “mutual information” [15]: 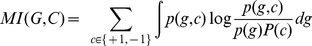
(Eq1)where *p*(*g*,*c*) is the empirical distribution of the gene expression *g* for the feature G over the patients whose ER-status is 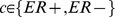, *p*(*g*) is the empirical distribution over all patients (of both classes), and *P*(*c*) is the empirical distribution of patients of the different classes – here, *P*(*C*  =  *ER*+) = 112/176 as 112 patients were ER-positive, and *P*(*C*  =  *ER*−) is 64/176. The distributions over continuous variables, *p*(*g*) and *p*(*g*,*c*), were estimated non-parametrically using Parzen Gaussian window, produced by the “maximum relevancy” component of the mRMR system [16].

### Feature-Selecting Support Vector Machine (for Machine Learning Analysis)

**Figure 1:**
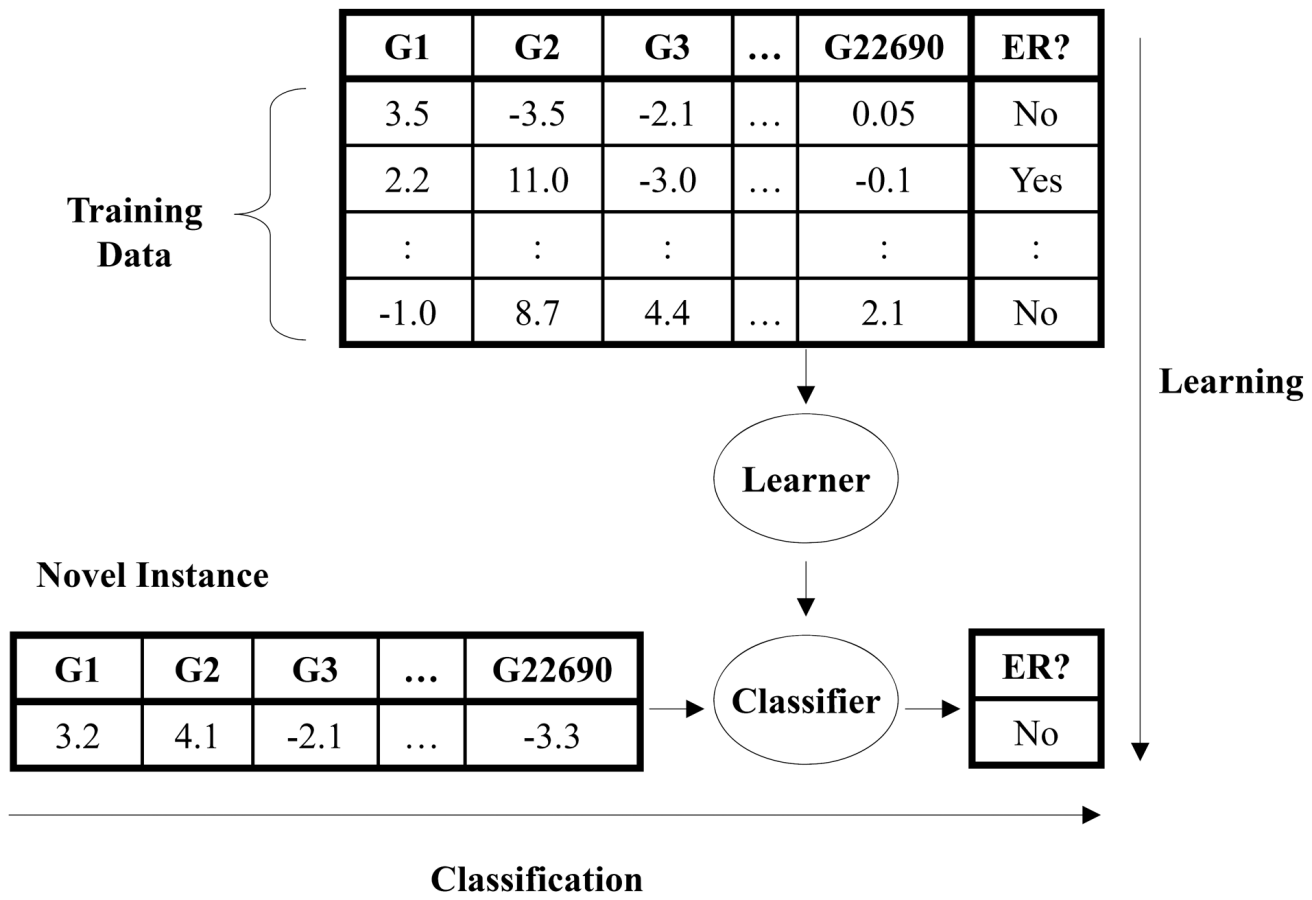
Basic machine learning framework. The bottom portion of this figures shows that a “Classifier” takes as input a description of a novel instance (here, the 27688 gene expression values from a microarray taken from a patient's biopsy), and returns a prediction for this instance (here, its prediction of whether this tumor is ER+ or ER−). The figure suggests this response is “No”. The Machine Learning challenge is to produce this classifier from a dataset of historical data (called labeled “Training Data”); this is the vertical portion, showing that a Learner uses that Training Data to produce the classifier. When evaluating the quality of a learned classifier, we require that the “Novel Instance” is not in the Training Data.

While the correlation of a single feature with a phenotype may be useful, it is not designed to predict whether a specific patient is ER+ versus ER−; for this, we need to use an alternative approach that can learn predictive combinations of multiple features. In particular, the machine learning approach uses this labeled dataset *D* (here E176, of gene expression values for 176 samples, each with known ER-status) to produce a classifier that can effectively predict the ER-status of a novel breast tumor (Figure 1). While this is similar to the biostatistics task, the goals are sufficiently different such that it requires this different technology.

**Figure 2:**
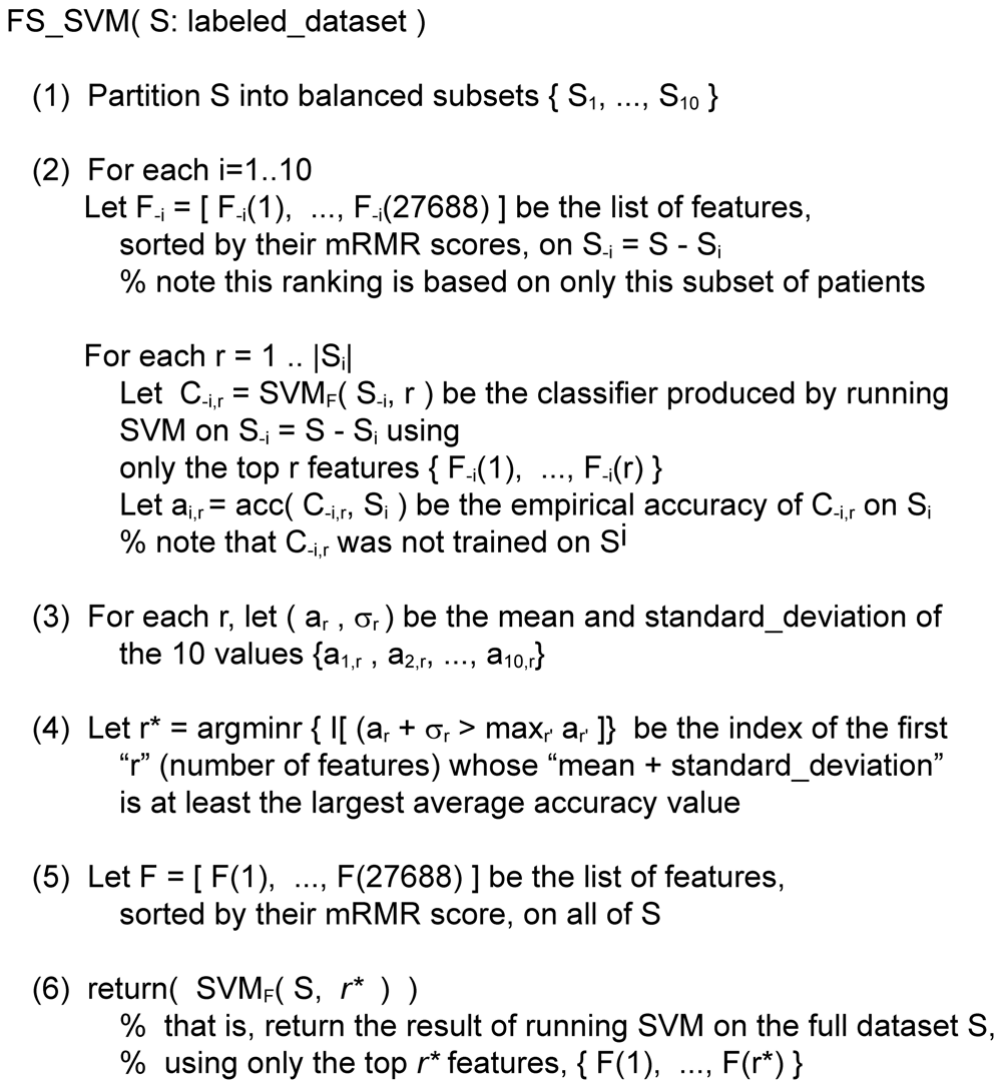
FS_SVM; a feature selection version of the Support Vector Machine (SVM) learner. Line 6 runs SVM on the dataset S, but uses only the r^*^ “best” features, where features are ranked by their mRMR score^15^, which is computed in Line 5. Note this mRMR score combines mutual information (Eq 1) with minimum redundancy. The goal of the first 4 lines is to compute this r^*^ value: Here, we first partition the dataset into 10 disjoint same-sized subsets {S_i_, _i = 1…10_}, which are balanced (ie, each is of the same size, and has about the same number of ER+ instances). FS_SVM then considers each of these S_i_ subsets, one by one. It first considers the remaining instances, S_−i_  =  S − S_i_, and computes the mRMR score for each feature with respect to this subset of instances. It then evaluates how well SVM does when using only the first r = 1, 2,… of these features, in order. Here, it runs SVM, using that size-r subset of features, on the training set S_−i_, then evaluates the resulting classifier on the remaining “testing subset” S_i_. Line 4 sets r^*^ to be the smallest value that is within 1 standard deviation of the high-water mark. See Material S1 for more details.

We considered several machine learning systems, before converging on the FS_SVM algorithm shown in Figure 2, which is a “feature selecting” variant of Support Vector Machines (SVM) [17], [18]. In general, the SVM learner uses a labeled data set to produce a linear separator between the classes – labeling a new patient, with microarray values 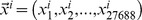, as 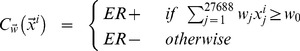
(Eq2)for some real-valued weight vector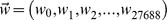; notice this vector includes the threshold 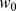. SVM learns the appropriate weights 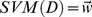 from the training sample *D* by attempting to optimize the margin [17]. The code was written in-house and used WEKA's SMO (with default parameters) for SVM [18]. To reduce the chance of overfitting, FS_SVM focuses SVM on only a small subset of the features: It first sorts the features based on their “Maximum Relevancy, Minimum Redundancy” (mRMR) [16] score on the E176 patients – this differs from the mutual information score (Eq 1) by finding the relevant features sequentially, and penalizing a feature by its correlation with features already included at any earlier stage. FS_SVM will use the top few of these features; it uses a variant of cross-validation to determine the smallest number that is statistically indistinguishable from the “high water mark” (see Material S1 for details of this algorithm).

### Graphing and Statistical Analysis

The graphical representations of the ER-status classifier and percent of patients with IHC-determined ER expression were generated with Microsoft Excel. Kaplan-Meier survival and recurrence-free survival curves and the Cox proportional hazards were generated in GraphPad Prism 5 using the Mantel-Haenszel approach [19].

## Results

This data (27,688 gene expression values over 176 breast cancer patients) can be analyzed using a biostatistical or a machine learning approach. The typical biostatistics approach is univariate, with the goal of finding the individual genes most correlated with the ER-status outcome. By contrast, the machine learning approach uses this data to produce a classifier, which can then be used to predict the ER-status of a novel breast tumor (Figure 1). While the learner has access to all 27,688 gene expression values, the classifier it produces will use only the genes that are necessary to achieve an accurate prediction. Notice these genes are not necessarily the ones that are individually most correlated with ER-status.

### Biostatistical Analysis

Using the gene expression values derived from 176 breast cancer samples (the E176 dataset), we found the 10 gene features that are most closely related to the ER-status of a tumor; ie, which individually had the highest mutual information (Eq 1) with ER-status. This list, shown in Table 1, includes many genes known to relate to estrogen receptor status in the context of breast cancer. Note that the estrogen receptor gene, ESR1, appears as the fourth entry. Genes whose expression is tightly regulated by ESR1 can also be correlated with the ER-status of a tumor. Some of these are known to be closely related to ER function, such as GATA3, while the relationship is not clear for others, like BCL11A. However, BCL11A is a zinc-finger protein, and other members of this class have been shown to regulate estrogen receptor expression in breast cancer cells [20].

**Table 1:** Top 10 genes, sorted by mutual information related to ER-status, based on the E176-cohort.

Index E176- cohort	Gene Name/Oligo ID	Mutual Information	FS_SVM Coefficients	Index E23 cohort	Gene Description
1	AW972815/A_32_P104334	0.8070	−0.2466	1284	human cDNA
2	GATA3/A_23_P75056	0.6497	2.2165	162	GATA binding protein 3
3	FABP7/A_23_P134139	0.6273		2182	fatty acid binding protein 7
4	ESR1/A_23_P309739	0.6262		22	estrogen receptor 1
5	CA12/A_23_P372234	0.6223	1.2934	76	carbonic anhydrase XII
6	BCL11A/A_24_P402588	0.6102		208	zinc-finger protein
7	BCL11A/A_24_P411186	0.5960		113	zinc-finger protein
8	CA12/A_24_P330518	0.5795		7	carbonic anhydrase XII
9	CYP2B6/A_24_P339514	0.5612		3	cytochrome P450
10	VGLL1/A_23_P253123	0.5532		662	transcription cofactor

This table also provides the SVM coefficient, the index over the E23-cohort (see text), and a short description of the gene.

### Machine Learning Analysis

**Figure 3:**
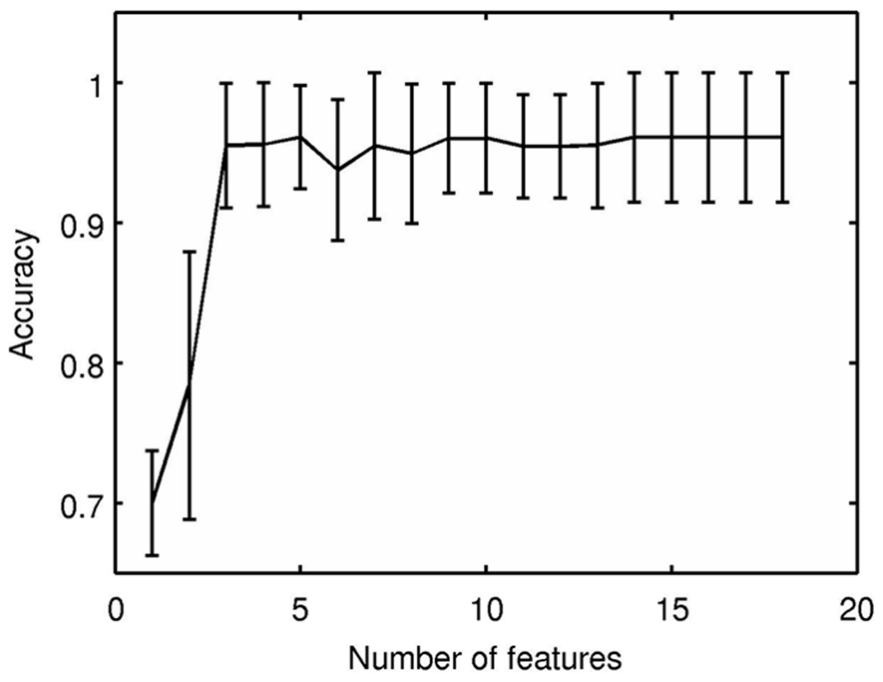
Average accuracy of SVM, as a function of number of features. For each r = 1,2,…,18, line 3 of FS_SVM (Figure 2) computes the mean *a_r_* and standard deviation *σ_r_* of the empirical accuracies obtained, over all 10 folds; this figure plots these 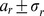 bars, for each r. Notice the average accuracy on the hold-out sets increases as the number of features is increased, then levels out, with only minor fluctuations. Here, the largest accuracy occurs at r = 4; notice however that this accuracy is “essentially” the same as at r = 3. We therefore set r^*^ = 3 as it is the smallest number of features whose accuracy's “mean + standard deviation” is at least the high-water-mark mean accuracy.

We also used the E176 dataset to learn a classifier, which can then be applied to predict the ER-status of any other tumor (Figure 1). Here, we used the FS_SVM learner shown in Figure 2. While FS_SVM had access to all 27,688 gene expression values, it produced a classifier that used only a small subset of the genes; just the three genes listed with entries in “SVM Coefficient” in Table 1: AW972815, GATA3 and CA12. FS_SVM determined that 3 genes were sufficient, based on the means and standard deviations associated with using only 1 feature, then 2 features, etc., across the folds (Figure 3 and Material S1). FS_SVM produced the following formula for predicting the ER-status of a novel tumor: 
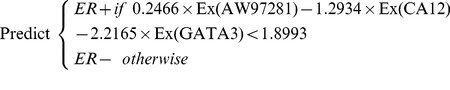
(Eq3)where Ex(g) refers to the gene expression value for the specified gene (g), for the current tumor. As noted above, the three gene features used by this classifier are not simply the top 3 features based on individual information (these features were ranked 1, 5, 2), but are instead the features whose mRMR scores are ranked highest (Table 1).

The quality of this classifier is how accurately it can predict the ER-status of a *novel* tumor. We initially estimated this using the 10-fold cross-validation accuracy of the FS_SVM learner applied to E176 [18], which was 93.17±2.44% – that is, around 6.8% error. This low error is within the range expected from gold-standard central laboratories, and is significantly lower than the 30% misclassification reported in one jurisdiction [3].

We then tested the validity of the classifier shown in Eq3 on the independent, novel cohort E23. These samples were normalized ([N1]–[N4]) independently of the E176 group, so the performance achieved with the E23 group is not dependent upon the E176 data itself, but only on the resulting Eq3 classifier. This classifier correctly labeled 22/23 = 95.65% on these patients, which is consistent with our cross-validation accuracy. This is more accurate than two other obvious classifiers: based on only a single gene, or on all of the genes (Material S2).

**Figure 4:**
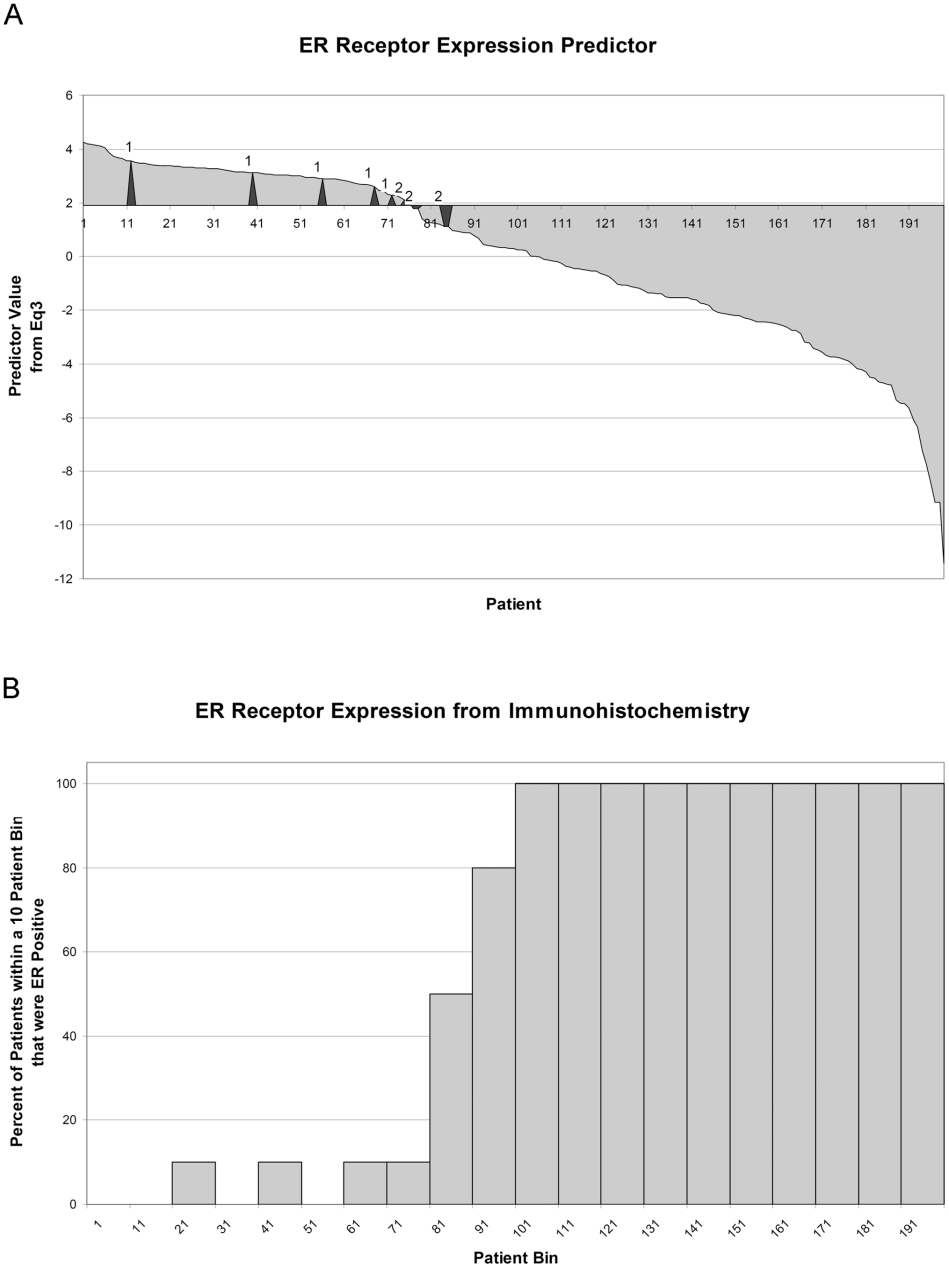
The Eq3 Classifier Predicts ER-Status with High Accuracy. The individual patient Eq3 values from the combined E176 and E23 datasets are sorted in descending order. The black triangular peaks mark patients classified as ER+ or ER- from IHC but the opposite from the Eq3 classifier, and the number of patients within each peak is labeled above. a) Histogram of the above sorted Eq3 values, showing the percentage of IHC-determined ER+ patients, in each 10-patient bin.

**Figure 5:**
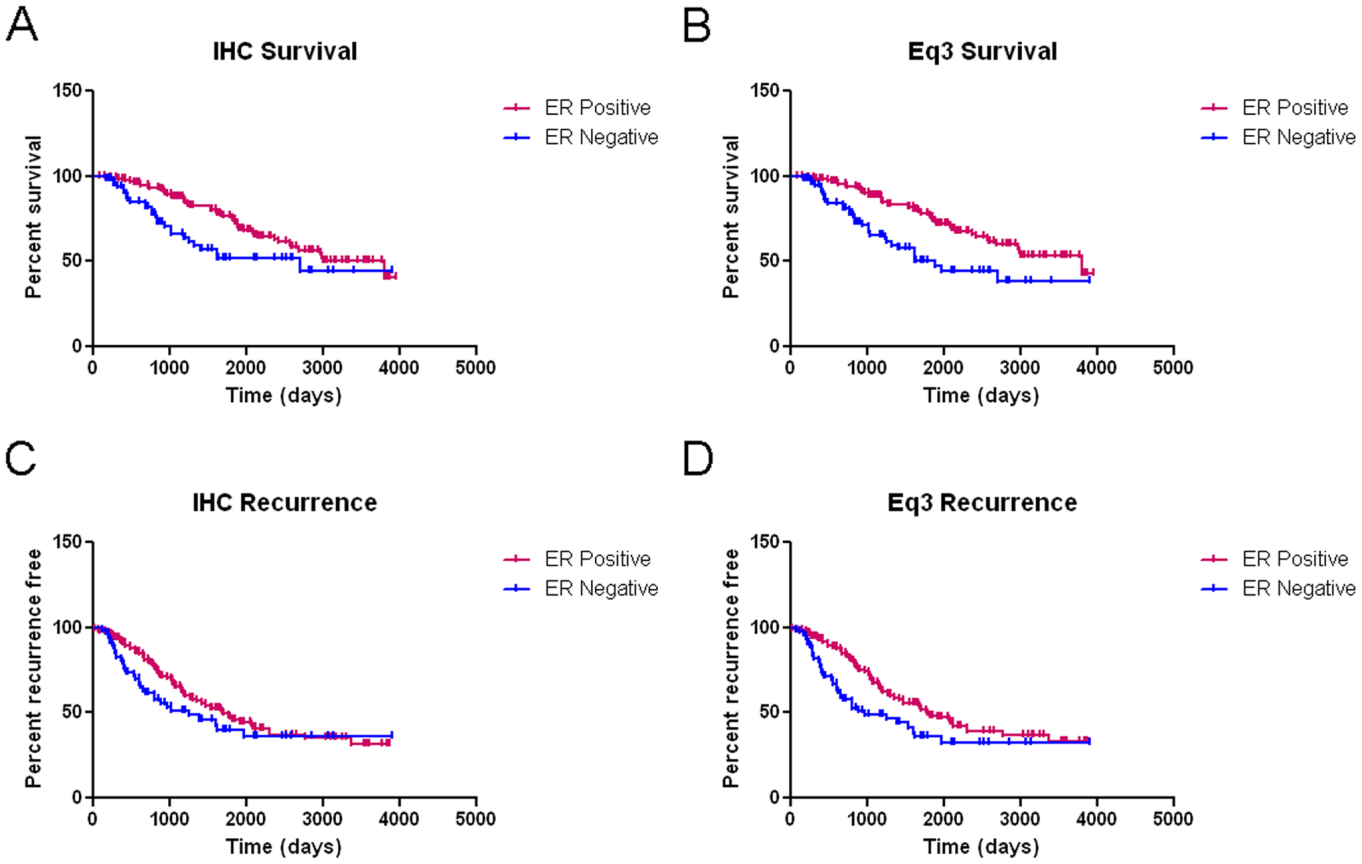
Kaplan-Meier Survival and Recurrence-Free Survival Curves For Patients Sorted by IHC-Determined ER-Status and Eq3 Predicted ER-Status. Both the survival and recurrence-free survival curves had greater separation and lower hazard ratios (HR) when the patients were sorted by Eq3 ER-status compared with traditional IHC. a) Survival curves for patients split based on IHC ER-status (ER+ n = 126, median survival = 3807days; ER- n = 72, median survival = 2704days; HR = 0.5090; 95% CI = 0.2968–0.8731). b) Survival curves for patients split based on Eq3 ER-status (ER+ n = 123, median survival = 3807days; ER- n = 75, median survival = 1623days; HR = 0.3901; 95% CI = 0.2420–0.6935). c) Recurrence-free survival curves for patients split based on IHC ER-status (ER+ n = 126, median recurrence-free survival = 1694days; ER- n = 72, median recurrence-free survival = 1246days; HR = 0.7160; 95% CI = 0.4623–1.109).d) Recurrence-free survival curves for patients split based on Eq3 ER-status (ER+ n = 123, median recurrence-free survival = 1820days; ER- n = 75, median recurrence-free survival = 875days; HR = 0.5731; 95% CI = 0.3718–0.8833).

Overall the Eq3 classifier correctly predicted 188/199 = 94.47% of the patients in this dataset. Of those misclassified, 4 were IHC ER- but predicted to be ER+ and 7 were IHC ER+ but predicted to be ER-. Closer examination of these misclassified patients revealed that, while some were close to the Eq3 cutoff value (7/11 are within 5%), others were much further away (Figure 4). For the 7 IHC ER+ patients that Eq3 predicted to be ER-, all 7 patients had early recurrence and 6/7 were deceased; for those 4 IHC ER- that Eq3 predicted to be ER+, only 1/4 patients had recurrence and was deceased from her cancer. Based upon this observation, we compared the survival and recurrence-free survival curves, and found that the curves based on Eq3-prediction had greater separation and lower hazard ratios than the ones based on IHC (0.4096 vs. 0.5090 for survival and 0.5731 vs. 0.7160 for recurrence-free survival) (Figure 5).

To further test the generality of our classifier, we next considered every other publicly-available dataset that specified the patient's ER-status, using the same Agilent platform (Table 2). For each of these data sets, we applied [N4] (transformed the data into z-scores), then used Eq3 to classify each instance. Our classifier correctly labeled 7/7 of the patients in the GEO dataset GSE26338 (5ER+ and 2 ER−); and correctly labeled 39/40 patients in dataset GSE5546 (30 ER+, 10 ER−).

**Table 2:** Accuracy for our 3-feature classifier, over various datasets.

DataSet	Platform	# ER+/ER−*	Accuracy of 3-Feature Classifier	Accuracy of Logistic Regression (Top 10 Oligos)
E176	Agilent	112/64	93.17±2.44% (10 fold CV)	
E23	Agilent	14/9	22/23 = 95.65%	22/23 = 95.65%
GSE26338	Agilent	5/2	7/7 = 100%	6/7 = 85.74%
GSE5546	Agilent	30/10	39/40 = 97.5%	23/40 = 57.5%
GSE19615	Affymetrix	72/43	108/115 = 93.91%	79/115 = 68.7%
GSE31448 (log)	Affymetrix	188/162	317/350 = 90.57%	188/350 = 53.71%

*#ER+/ER−: Number of patients that were estrogen receptor positive/negative from gold standard IHC analysis.

We then sought publicly-available datasets specifying ER-status, on other platforms; this identified two Affymetrix datasets (Table 2). We used BLAST [21] to correlate the sequence of each relevant Agilent probe to the closest matches in the Affymetrix probes: A_32_P104334 (AW97281) matches perfectly to 230356_at; the closest match to A_23_P372234 (CA12) is 203963_at; and the closest match to A_23_P75056 (GATA3) is 209602_s_at.

Dataset GSE19615 has 115 samples (72 ER+, 43 ER−). We first transform the data using z-score [N4] and then apply Eq3 to the data. The classifier correctly labeled 108/115 samples (93.9% accuracy). Dataset GSE31448 has 350 samples with known ER-status values (188 ER+, 162 ER−). Since the normalization of this data was different than ours and the other datasets that we used, we had to first exponentiate its values (to be in the same range as other datasets), before applying the z-score transform [N4]. Applying Eq3 to this data correctly labeled 317/350 samples (90.6% accuracy). We also considered using the more standard approach, of simply computing the best classifier based on top 10 features. As such we learned a logistic regression classifier using the top 10 genes identified in the E176 dataset. However, in the external datasets, the accuracy of this resulting classifier ranged from only 53.71% to 85.71% (Table 2).

Three of these four publicly available datasets also had recurrence data. For the 2 Agilent datasets, only one patient was classified differently by our classifier; IHC claimed she was ER+ while our classifier predicted ER− and this patient experienced recurrence. Of the 7 misclassified patients in the GSE19615 dataset, only one had recurrence. This resulted in only small differences in the hazard ratios between IHC and Eq3-classified ER-status and recurrence-free survival.

The above results were based on applying Eq3 to the normalized expression values of only three genes. Note, however, that step N2 of the normalization procedure relied on information based on the expression values of other genes in the microarray, and steps N3 and N4 relied on having access to a large number of patients. To explore whether we could make predictions for an individual patient, using only a small number of expression values (rather than the full microarray), we considered the simpler normalization approach of simply log-transforming the expression values of these three genes, and subtracting the log of the expression of the “housekeeping” gene ACTB (beta-actin) (oligo A_23_P135769). This produced slightly modified E176′ and E23′ datasets, that used only these 4 values per patient. We then ran the SVM learning algorithm to this E176′ data, and found this produced a 10-fold cross-validation accuracy of 162/176 (92.045% accuracy); this classifier then correctly classified 22/23 correctly (95.65% accuracy) on the E23′ dataset.

## Discussion

Breast cancer transcriptome analyses have been performed for a variety of purposes. Several studies of frozen primary breast cancers were designed to find a set of genes whose expressions are most correlated with new molecular sub-classifications of the disease [4], [6], [22]–[37], or to provide prognostic algorithms related to risk of relapse and death [28], [31], [32].

Some of the published studies are “focused”, in that they examine just a few specified genes with established relationships to the disease phenotype [24], [32], [38]. One limitation of those focused studies is that they require prior knowledge about the disease that, if incomplete or incorrect, means they will not use relevant high-performing biomarkers. Our work is at the other extreme: we began with a transcriptome-wide set of all genes [4], [34], [39], [40], from which we sought transcription patterns that relate to the patient phenotype, without prior specific biological understanding nor pre-specified hypotheses.

Our goal was to address a specific unmet medical need: to generate a simple, RNA-based classifier of breast cancer ER-status that would be amenable to high-throughput, objective analysis of formalin-fixed, paraffin-embedded tissue. While we used fresh frozen tissues to perform our analysis here, such tissues are neither generally available nor suitable for routine clinical analysis, which is why most clinical decisions are based on formalin-fixed, paraffin-embedded tissues. Due to the technical limitations of analysis of RNA in fixed tissues, it is desirable to use a small number of transcripts, to facilitate quantitative RT-PCR based assessments of fixed materials, as is done in the commercially available Oncotype DX platform [41]. Table 3 provides a short comparison, highlighting the major advantages and disadvantages of these different technologies.

**Table 3:** Comparison of the advantages and disadvantages of IHC versus gene expression for tumor assessment (e.g. ER-status).

	Immunohistochemistry		Gene Expression	
Issue	Advantage	Disadvantage	Advantage	Disadvantage
**Specificity**	Specific to malignant cells	Restricted to known proteins	Not restricted to known/prespecified features; allows large “discovery set” evaluation	Stromal contamination unless microdissection
**Target**	Protein based	Subjective interpretation (not yet amenable to automated interpretation; only malignant cells scored)	Objective and quantitative interpretation	Technically challenging to apply to fixed tissues
**Preanalytic Variables**	Does not require frozen tissue; routinely applied to fixed tissues	Preanalytic variability (fixation time and method) cannot be readily assessed)	Effects of preanalytic variability can be assessed with RNA quality; potential for automated interpretation	Preanalytic variability (RNA quality)

We have shown that machine learning analysis of 27,688 transcription values can be used to generate a simple classifier that uses transcription values of only three genes to reliably and efficiently classify novel, independent primary breast cancer ER-status, with approximately 93% accuracy. Moreover, this classifier is extremely robust as it is able to correctly classify patients from different studies, produced on different platforms in different labs, with the same high accuracy. To our knowledge, this is the only study that has shown that a model learned from one platform, can work effectively on another. It also appears to work effectively using only the expression value of a very small set of genes – i.e., it does not require a full microarray.

As noted above, the goal of the machine learning system, to produce a classifier, is different from the goal of the standard biostatistics approach, to find the features most relevant to a phenotype, here ER-status. This leads to another distinction: while the results of the biostatistics approach (a specific set of biomarkers) often varies significantly across datasets [42], the results of the machine learning approach (here, a classifier) typically does not vary (Table 2). The literature includes many examples where one dataset suggests that one set of genes is relevant with respect to a phenotype, but another dataset suggests a very different set of genes for the same phenotype. As one example, both Sorlie *et al.* [34] and van't Veer *et al.* [7] sought genes related to survival of breast cancer patients, identifying sets of 456 genes and 231 genes respectively. Unfortunately, these two sets have a relatively small intersection, with Jaccard score (intersection/union) of only 0.025 [43].

We found that the association sets on our two datasets were similarly inconsistent. Table 1 shows the 10 genes that the E176-cohort considers most relevant to ER-status. The “Index (23-cohort)” column shows that the E23-cohort places only 2 of these in its top-10, corresponding to a Jaccard score of only 2/18≈0.11. Note also that only 4 of these E176 top-10 are in the E23 top-100, and only 24 of the E176 top-100 are in the E23 top-100 (Jaccard of 24/176≈0.136). While this problem is standard for association studies that deal with different datasets, it is not a problem for our (machine learning) prediction study, as we found that the classifier, based on the E176-cohort, was extremely accurate on the independent E23-cohort validation set, as well as 4 other publically-available datasets, including two using a different platform. This is because the goal of a learning system is different, in explicitly seeking a classifier, which applies in general and in particular, designed to correctly classify novel subjects (i.e., patients who were not in its training set).

One potential problem of this study is that we are comparing the results of the 3 gene classifier against IHC results, which might have misclassified the patient ER status. The misclassified patients in our dataset had clinical outcomes more closely correlated with their predicted ER-status than their IHC-determined ER-status. This may indicate that this classifier is a better predictor of survival and recurrence than IHC ER-status and may reflect the activity of the estrogen receptor rather than just its expression, but further work is needed to confirm this.

Another advantage of our classifier is that it only uses three features, which is significantly fewer than the 550 genes used by the classifier produced by Van't Veer *et al*. [7]. Many other researchers have used various machine learning methods (Artificial Neural Networks, Weighted-Voting, SVM, Logistic Regression [6], [29], [44] to produce classifiers for predicting ER-status; each of these classifiers similarly required a large number of genes (5000 to 25000) for this prediction task. Additional discussion of related studies is available in Material S3.

The strength of our classifier is further demonstrated by the fact that the 3 features, normalized against a single housekeeping gene, were able to classify the E176 and E23 datasets with 92.045% and 95.65% accuracy respectively, indicating that it may be possible to develop a test for ER-status with as few as four genes; of course, future validation is required.

## Conclusion

Effective management of breast cancers relies heavily on accurately determining the tumor's ER-status. While standard IHC assessments are reasonably accurate, they are subject to human error, are dependent on pre-analytic variables, and lack robust internal positive and negative controls. We therefore propose a transcription-based assessment for ER-status, and find that a learned combination of the assessment of 3 specific genes is sufficient to classify ER-status with approximately 93% accuracy in both a 176-patient training cohort, and also in several independent datasets, including some from different RNA-based platforms.

Given the many methodological advantages of the FS_SVM learning algorithm, we believe that this learning tool has general applicability, in that it produces a classifier that uses a small subset of features to reliably predict a phenotype. Future prospective studies with qRT-PCR of these 3 genes and beta-actin for normalization will determine if this classifier is a better predictor of endocrine therapy response than the current assessment methodology. Given the inherent variability of many IHC diagnostic tests, this approach warrants further evaluation in the setting of cancer biomarker discovery and validation.

## Supporting Information

Material S1 **The FS_SVM Algorithm.**
(DOC)

Material S2 **Results using other Classifiers**.
(DOC)

Material S3 **Further Discussion of Related Studies.**
(DOC)
