# Supplementary material for: A Machine Learned Classifier That Uses Gene Expression Data to Accurately Predict Estrogen Receptor Status
Source: PLoS One. 2013 Dec 2;8(12):e82144. doi: 10.1371/journal.pone.0082144 (PMC3846850; doi:10.1371/journal.pone.0082144)
Supplement: Material S1 — The FS_SVM Algorithm. (DOC) [file pone.0082144.s001.doc]

**Supplementary Material S1**

**The FS_SVM Algorithm:**

This supplementary material will motivate the feature-selecting support vector machine (FS_SVM) algorithm – a tool for learning a classifier from very high dimensional data.

FS_SVM uses linear SVM, which is a standard machine learning tool that takes a data set and returns a classifier – see Figure 1. The resulting classifier is a linear separator over all of the variables; see Eq 2. Hence, running SVM itself on the data will produce coefficients for all 27,688 genes, which means the final assessment for a patient will depend on the expression x_i_ of each of her genes. This is biologically problematic, as it implicitly suggests that all of the genes are somehow relevant. Another problem is "overfitting": It is easy to express a huge number of (linear) functions when using these 27,688 +1 = 27,689 parameters, which means it is easy to find the parameters that nicely match the training sample. In fact, this task is "too" easy, meaning that the function produced here is likely to do poorly on novel patients.

One way to address both problems is to use only a subset of relevant genes. A naïve way to do this is just to consider various subsets, and use the subset that does best over the training data. Unfortunately, this is problematic [1-4].

To explain the issues, we need to use the following notation: For any classifier *C*, the quantity we really want to maximize is *C*’s true accuracy, denoted *acc(C),* which is the probability that a patient, drawn from the distribution of patients who are being considered (e.g., that have breast cancer), will be correctly diagnosed: that is, an ER+ patient is correctly predicted to be ER+, and an ER- is correctly predicted to be ER-. Unfortunately, we cannot compute this, as it depends on the underlying distribution of instances, and their true labels, which we in general do not know. Given a labeled dataset *S* = { }, we can, however, compute the empirical accuracy obtained when testing the classifier *C* on the dataset *S*: (The “indicator” function "*I* [a=b]" is 1 if a=b, and is 0 otherwise.)

Now let *C­_D_ = L(D)* be the classifier obtained by applying the learning algorithm *L*(.) to the dataset *D*. One (problematic) way to estimate this error is to evaluate this C_D_ classifier by applying it to the current training dataset *D* (i.e. on the dataset from which it was learned), and measuring how often this classifier predicted the incorrect answer -- i.e. using $\hat{a}\left(C_{D},D\right)$. However, this measure does not give meaningful answers, as it is using the training data to evaluate the result. (This is like allowing students to train on the actual final exam, before later testing them on that exact same examination; their scores will be higher than their true knowledge.)

A better approach to estimating *acc(C_D_) –* the quality of this *C_D_* – is using *k=10* fold cross-validation [5]. Here we first partition *D* into *k* disjoint subsets $D=D_{1}\bigcup D_{2}\bigcup...\bigcup D_{k}$ (which are balanced -- i.e. each is about the same size, and also has about the same number of ER+ instances), and let $D_{-i}=D-D_{i}$ be the subset that does not include$D_{i}$. Now for each *i=1..k*, let $C_{i}=L\left(D_{-i}\right)$ be the classifier obtained by applying *L*(.) to the *D_-i_* subset of the original labeled data, then compute the accuracy of this classifier on the remaining instances $D_{i}$-- i.e. compute $\hat{a}\left(C_{-i},D_{i}\right)$. The average over the *k* values  is a good estimate of the quality of the classifier obtained by training *L*(.) on the complete *D* – that is, . Notice each patient is tested exactly once. This so-called "*k*-fold cross validation" is used to estimate the quality of our learned classifiers [5].

FS_SVM, a feature selection version of the Support Vector Machine (SVM), will run SVM on a small subset of the features, and will use an idea related to cross-validation to determine the size of this subset (i.e., the appropriate number of features). As shown in Figure 2, FS_SVM’s Line 6 runs SVM on the dataset *S*, but using only on the *r^*^* "best" features, where features are ranked by their mRMR score [6], which is computed in Line 5. (Note this mRMR score combines mutual information (Eq 1) with minimum redundancy). Here, we want *r^**^ = argmax_r_ (acc( SVM(D,r) ) )*, where *SVM(D,r)* is the classifier produced by running SVM on the complete dataset *D*, but only using the top r^*^ features of each instance (where “top” is based on the mRMR-score of each feature, on all of *D*). We can estimate r^**^ as $r^{(*)}=\mathrm{argmax}_{r} \{ \hat{a}\left( SVM(D,r), D '\right) \}$ where *D’* is a disjoint from *D*, but drawn from the same distribution. As we do not have a separate *D’* set, we could use simply $r^{+}=\mathrm{argmax}_{r} \{ \hat{a}\left( SVM(D,r), D \right) \}$. Of course this is problematic, as the *SVM(D,r)* classifier was learned on *D*, and so its empirical accuracy on *D* will be biased. Moreover, the “bias” might not be a constant across the value *r*’s – i.e., $\hat{a}\left( SVM(D,3), D \right)$might be bigger than$\hat{a}\left( SVM(D,5), D \right)$, even though $acc\left( SVM(D,3) \right) < acc\left( SVM(D,5) \right)$, which means this *r^+^* might be very different from the true *r^*^*.

FS_SVM instead uses the “cross-validation” trick, of first partitioning the dataset *D* into 10 disjoint balanced subsets { *D_i_* }. It then considers each of these *D_i_* subsets, one by one. It first computes the mRMR score for each feature with respect to *D_i_*’s complement, *D_-i_ = D - D_i_*, then evaluates how well SVM does when using only the first r=1, 2, ... of these features, in order. Here, it runs the SVM learner using only that size-*r* subset of features, on the training set *D_-i_*, then evaluates the resulting classifier on the remaining “testing subset” *D_i_*, $\hat{a}\left( SVM(D_{-i},r), D_{i} \right)$. For each r, it computes the mean (which is an approximation to $\hat{a}\left( SVM(D,r), D '\right)$) and variance over the *k*=10 instances. This information appears in Figure 3.

Now observe that *a(r; D)* increases for *r*=1,2,3 but then levels off; in particular, we see that the values are *{ a(3; D) , a(4;D), …, a(18;D) }* are all essentially the same value. This motivates us to set *r^*^* to be the smallest value that is within 1 standard deviation of the high-water mark; see Line 4 (Figure 2).

Note that FS_SVM runs these k=10 folds to compute *r^*^*, which means it calls SVM 10 times to compute *r^*+^*, followed by one more call to compute the actual classifier that it returns. To estimate the quality of the resulting classifier, we also run this entire FS_SVM 10 more times. Hence, to return both the classifier and estimate of its accuracy, we will call SVM (10+1)×(10+1) =121 times, over various subsets of the data. As noted above, this produces the classifier shown in Eq3, with a cross-validation accuracy of 93.17 ± 2.44%.

**References:**

1. Dupuy A, Simon RM (2007) Critical review of published microarray studies for cancer outcome and guidelines on statistical analysis and reporting. J Natl Cancer Inst 99: 147-157.

**2. Molla M, Waddell M, D. P, J. S (2004) Using Machine Learning to Design and Interpret Gene-Expression Microarrays. AI Magazine 25: 23-44.**

**3. Page D (2007) SIGKDD Webinar on Mining High-Throughput Biological Data.**

**4. van 't Veer LJ, Dai H, van de Vijver MJ, He YD, Hart AA, et al. (2002) Gene expression profiling predicts clinical outcome of breast cancer. Nature 415: 530-536.**

**5. Witten IH, Frank E, Hall MA (2011) Data mining : practical machine learning tools and techniques Burlington, MA: Morgan Kaufmann. 629 p.**

**6. Peng H, Long F, Ding C (2005) Feature selection based on mutual information: criteria of max-dependency, max-relevance, and min-redundancy. IEEE Trans Pattern Anal Mach Intell 27: 1226-1238.**
