# Supplementary material for: A Machine Learned Classifier That Uses Gene Expression Data to Accurately Predict Estrogen Receptor Status
Source: PLoS One. 2013 Dec 2;8(12):e82144. doi: 10.1371/journal.pone.0082144 (PMC3846850; doi:10.1371/journal.pone.0082144)
Supplement: Material S2 — Results using other Classifiers. (DOC) [file pone.0082144.s002.doc]

**Supplementary Material S2**

**Results using other Classifiers**:

We also considered two other possible classifiers. One used just the mostly highly correlated single feature (which is "AW972815" in 9 of the 10 folds); here we achieve an accuracy of only 70.04 ± 3.8%, which is significantly inferior to our FS_SVM classifier, which is allowed to use several features. Each of the learners mentioned here is evaluated using the same 10-fold cross-valuation described above, using the same folds. This allows our statistical evaluations to be 2-sided paired t-tests. Throughout, we declare a difference to be significant if it rejects the "same distribution" null-hypothesis at the p<0.05 level.

Using the other extreme, of all features is similarly unhelpful: while the cross-validation results here on the 176-cohort seem comparable to the results using FS_SVM (94.96%; see “Standard SVM” entry for E176 row in Table 2), the real results, on the 23-patients hold-out cohort (E23 row) is significantly inferior here (only 89.96%).
