# Supplementary material for: A Machine Learned Classifier That Uses Gene Expression Data to Accurately Predict Estrogen Receptor Status
Source: PLoS One. 2013 Dec 2;8(12):e82144. doi: 10.1371/journal.pone.0082144 (PMC3846850; doi:10.1371/journal.pone.0082144)
Supplement: Material S3 — Further Discussion of Related Studies. (DOC) [file pone.0082144.s003.doc]

**Supplementary Material S3**

**Further Discussion of Related Studies:**

Our results also compare favorably with other published literature on evaluating transcriptomic profiles as correlates of ER. In a highly focused study, Gong *et al.* tried to predict the ER-status of tumors using only oligos that are already known to be related to the ESR1 (Estrogen Receptor Alpha) gene [1]. They found 9 probe-sets that aligned to different locations of the ESR1 mRNA, selected the single probe-set most correlated with ER-status, and calculated an appropriate threshold to predict the ER-status for future patients. While this produced accuracies ranging from 88 to 96% on two validations sets, the higher result was seen in a validation set with an uncharacteristically high rate of ER positivity (85% -- which means their result was only ~3% above baseline) and in which 40% of the samples returned “no call”. Our analysis used data from patient cohorts with a more clinically relevant proportion of ER negativity; moreover, our classifier returned a prediction for *all* of the patients (i.e., we did not produce any “no calls”). While focused studies require prior knowledge about the genes that are related to the disease, our learning method did not require such prior biological information, allowing our learner to produce a predictor that can include genes not previously known to be associated. Van't Veer *et al.* predicted ER-status by first selecting the 550 genes most correlated with ER-status on the entire data, then building a classifier using just those genes; it appeared to obtain a Leave-One-Out cross-validation accuracy of 90% [2]. However, that result is problematic, as it used all of the training data when selecting gene features, which led to overly-optimistic estimates of performance [3]. That study highlights the difficulties in accurately accessing the true accuracy of a learned classifier; much of the complexity of our FS_SVM algorithm (Figure 2) is designed to avoid this pitfall [4,5].

**References:**

1. Gong Y, Yan K, Lin F, Anderson K, Sotiriou C, et al. (2007) Determination of oestrogen-receptor status and ERBB2 status of breast carcinoma: a gene-expression profiling study. Lancet Oncol 8: 203-211.

2. van 't Veer LJ, Dai H, van de Vijver MJ, He YD, Hart AA, et al. (2002) Gene expression profiling predicts clinical outcome of breast cancer. Nature 415: 530-536.

3. Molla M, Waddell M, D. P, J. S (2004) Using Machine Learning to Design and Interpret Gene-Expression Microarrays. AI Magazine 25: 23-44.

4. Dupuy A, Simon RM (2007) Critical review of published microarray studies for cancer outcome and guidelines on statistical analysis and reporting. J Natl Cancer Inst 99: 147-157.

5. Smialowski P, Frishman D, Kramer S (2010) Pitfalls of supervised feature selection. Bioinformatics 26: 440-443.
